# Hospital and Institutional News

**Published:** 1919-12-20

**Authors:** 


### HOSPITAL AND INSTITUTIONAL NEWS.

### COLONEL BRUCE-VAUGHAN'S SUCCESSOR.

Sir William James Thomas, Bart., has been appointed to succeed the late Colonel E. M. Bruce-Vaughan as deputy-chairman of King Edward VII. Hospital, Cardiff. At the meeting at which this appointment was made the report of the sub-committee upon the reorganisation of the staff was submitted, but eventually referred to the Emergency Committee, so that every member of the board might become familiar with the scheme. But the general principle was accepted. We hope that Sir William James Thomas will lend his support to all proposals which will help to free the general superintendent, Mr. Rea, from mere routine, since the financial policy must depend for its success on the freedom with which he and Mr. Rea are provided to initiate ways and means. Publicity and appeals, in the sense of using every opportunity to attract interest and to enlist support, must be a daily, not a sporadic pre-occupation in Cardiff. To make full use of every chance, Sir W. J. Thomas and Mr. Rea should play into one another's hands, and continue or rather develop the policy of Colonel Vaughan which made the doings and needs of King Edward VII. Hospital a familiar household concern to every family and newspaper in Cardiff.

### SPIRITUALISM AND THE PROFESSION.

Sir Arthur Conan Doyle lectured a short time ago to a medical audience at a crowded meeting of the St. Mary's Hospital Medical Society. His subject was "The Material Side of the Psychic Movement." Speaking as a medical man to a medical audience, he said there was no objection which could occur to the most sceptical which had not in the past occurred to him. But he could not believe that men of honour, such as Crookes and others, could be mistaken in the evidence they had adduced. Spiritualism, Sir Arthur said, was too big a thing to be a religion. It was part of the common heritage of mankind. He was at one with the churches in combating materialism.

### DINNER TO SIR CHARLES CAMERON.

Few medical men, and even fewer eminent ones, live to their ninetieth year in full possession of their faculties. It is not surprising, therefore, that the Irish College of Surgeons entertained this well-known sanitarian on the occasion of a birthday of such high figure. Sir Charles, in one of those humorous after-dinner speeches for which he is noted, declared that the freedom of Dublin and the presidency of the college had by no means satiated his appetite for honours. He compared the present death-rate of the city with what it was many years before; in spite of the effects of the war, a significant decline has occurred. Students of heredity may be interested in Sir Charles' account of his birth and parentage: he was the child, he declared, of an Irishwoman and "a Gaelic-speaking Highlander." We may be excused the remark that, in spite of this, his personality shows few indications of the Celtic melancholy Matthew Arnold described. All medical men will wish this merry veteran smooth progress towards his centenary.

### AN INTERESTING APPOINTMENT.

We are happy to record that Mr. Phillips Franklin, F.R.C.S., has been appointed Surgeon-in-Charge of the Throat and Ear Departments of the East London Hospital for Children. Mr. Franklin is responsible for much of the organisation of the new American Hospital in London, and is himself an American citizen. His new appointment will undoubtedly form yet another link in the Anglo-American medical *entente*, which he and others have laboured so earnestly to promote, and one of the first fruits of which is the "Fellowship of Medicine." Mr. Franklin is hon. secretary of this latter body, and we congratulate him on the skill with which he has conducted its affairs.

### SCARLET FEVER IN LONDON.

The past two weeks have been particularly black with regard to the scarlet fever and diphtheria problems in London. For the former disease, for the week ending November 29, the figures were 2,490; for the week ending December 6, 2,989; and the deaths have increased for the latter week. Diphtheria incidence has run in an almost exactly parallel fashion, deaths having reached a weekly figure of 28. We read that the accommodation of the hospitals of the Metropolitan Asylums Board has been strained to its utmost; from one locality it being reported that relatively few patients could now be moved from their homes, all beds being taken up and fresh cases admitted only as those recovered were discharged. In one day 119 fresh cases were taken in. There is, as we have previously explained, nothing very unusual in the increased incidence of these infections in the latter half of the year, but, nevertheless, the present is undoubtedly an exceptionally severe outbreak. No explanation has as yet come to hand of the exact origin, whether by "carrier" or infected milk supply, although we understand that the Ministry of Health is actively engaged in the investigation.

### THE NEW SCOPE OF MEDICAL TEACHING.

The new professor of preventive medicine at the University College of South Wales and Monmouthshire has delivered his inaugural address, and the occasion is important in the growth of the Welsh Medical School. Dr. E. L. Collis, technically the Mansel Talbot Professor-Designate, spoke on the "aims of the Welsh national school of medicine." "Hospital medical schools," he said, "are more utilitarian, and the older university schools more scientific, but there has lately arisen the new science of preventive medicine which touches the life of the community and is neither purely scientific nor utilitarian in the sense implied by private practice. Preventive medicine has been official. It should become more personal, and sanitation is becoming more interesting through the activities of the school doctor and the welfare centre. As clinical medicine discovered the trained nurse, so preventive medicine invented the health visitor. The Welsh national medical school has to provide a trained medical service; co-ordination and not mere proficiency must be its aim." The lecture was a happy inauguration of a new school, which should influence the scope of medical training in other centres.

### INCREASED PAYMENTS FOR PENSIONER PATIENTS.

A deputation of the British Hospitals Association waited upon the Ministry of Pensions recently with a view to discuss a possible revision of the rates paid by the Ministry for the treatment of patients in voluntary hospitals. Following upon this action it is announced that increased rates, subject to the sanction of the Treasury, will come into force on January 1, 1920. It will be necessary, however, for hospitals asking for consideration to apply before that date in order that any increased grant made may be retrospective to the first day of the year. In respect of in-patients it is proposed that there shall be a sliding scale up to 11*s*. per day, according to the circumstances of the hospital and the cost of maintenance. For out-patients the existing flat rate of 2*s*. per attendance shall be continued, and, in order to encourage the institution of evening clinics the Ministry will guarantee a minimum total payment of two guineas per session per clinic, irrespective of the number of attendances. Higher rates are possible in respect of aural and ophthalmic clinics. The British Hospitals Association meet on January 15, 1920, to discuss the general financial position of voluntary hospitals.

### A MEDICAL CONFERENCE.

It is satisfactory to note that at the medical conference held by the British Federation of Medical and Allied Societies in London on December 12 the attitude was generally one of conciliation and of a general desire to work in harmony with the authorities towards general public health improvement. It was, however, clear that the medical profession intend to demand certain fundamental rights, and the following resolution was carried:—"That in the opinion of this conference . . . the draft regulations for 1920, proposed to be made by the Minister of Health with respect to the administration of the National Health Insurance medical benefit, are not adapted to secure the more efficient services from the medical profession nor adequate results to the community." It was therefore decided to ask the Government to set up a committee of inquiry and to convey their resolutions to the Prime Minister.

### A HARDSHIP TO ACCIDENT CASES.

Dr. Lionel Baly, medical superintendent of Lambeth Infirmary, has drawn the attention of the authorities at King's College Hospital to the admission of a patient from the hospital to the infirmary suffering from the effects of a fractured femur, which occurred a fortnight before he came under Dr. Baly's care. He pointed out that it is prejudicial to patients of this kind to be transferred from one institution to another when their treatment is incomplete, and that it would be far better for patients suffering from the results of accidents to be sent direct to the infirmary rather than to be taken into the hospital temporarily. Only patients resident in Lambeth should be sent to this infirmary. King's College Hospital, he added, is in a very unfortunate position for receiving accident cases occurring in four adjacent boroughs. Frequently the officers in charge of accident cases remove them in the London County Council ambulances from the hospitals, such as St. Thomas's, to Lambeth Infirmary, which is thus liable to receive patients from Battersea, Southwark, and other boroughs. When these patients arrive, the Medical Superintendent has no option but to take them in. The guardians have empowered their clerk to interview the chief officers of the London County Council ambulance service to ascertain if some other arrangement cannot be made.

### SOMETHING LIKE MUNICIPAL ENTERPRISE.

In its new Parliamentary Bill the Salford Corporation proposes to seek a comprehensive series of new health powers, including the provision and maintenance of ash-bins; regulations regarding the air space for habitable rooms and the minimum number and size of living- and bed-rooms in new dwelling houses; restrictions on the height and projection of wing buildings; the provision of larders in domestic buildings; the provision of closet accommodation in houses occupied by more than one family; the abatement of nuisances in private gardens; the making of by-laws in regard to dairies, milkshops and premises used for the cooking, sale and preparation of food; regulations as to covering foodstuffs during conveyance through streets; the cleansing of houses infected with vermin; the provision of accommodation for cleansing verminous persons; the prevention of the spread of infectious disease; the payment of compensation to persons ceasing their employment to prevent the spread of infectious disease; the removal to hospital of persons suffering from tuberculosis and the making good of any loss thereby sustained by such persons; making by-laws prohibiting the sale of certain meat and the prohibition of the use of inedible fats in food. Prodigious! but the fact is that all these powers are already in existence in different parts of the country. They are only surprising when in bulk, and the marvel is that when one corporation gets valuable health powers Parliament does not automatically and simultaneously give them to all authorities.

### MR. COLERIDGE HARPER'S PROPOSAL.

A practical suggestion for remedying the shortage of accommodation for lying-in cases has been made by the Rev. Coleridge Harper, rector of St. Mary's, Newington. Writing to the Southwark and Lambeth Boards of Guardians on behalf of the local Baby Care Committee he states that, because of the number of returned soldiers and recent marriages, there is an immediate prospective increase of births. In consequence, the lying-in hospitals are overcrowded. The alternative for the patients is the infirmary, and the guardians may realise the rooted objection of the respectable poor to enter the infirmaries. Would the guardians consider reserving part of their infirmaries under a special name, for the use of such mothers on conditions similar to those at the Lying-in Hospital, York Road, Lambeth? In both instances the suggestion was sympathetically received, and was referred to the committees for consideration. There are, of course, legal difficulties to surmount under the existing Poor Law, but, as there is an apparent disposition to remodel the whole fabric of Poor-Law administration, the Rev. Coleridge Harper's proposal should be carefully considered.

### THE PHYSICIAN'S CANE.

A correspondent reports a somewhat interesting note relating to the old custom among physicians of carrying a cane. In the College of Physicians, he writes, is to be seen a gold mounted cane which in its day passed through the hands of no fewer than six distinguished doctors. Originally it belonged to John Radcliffe, Fellow of the College of Physicians in 1687, who bequeathed £500 to St. Bartholomew's for "mending the diet" of the patients; Radcliffe gave it to Mead, the author of "The Power of the Sun and Moon over Human Bodies," who in turn passed it on to Askew, from whom it descended successively to William and David Pitcairn, the latter presenting it to Matthew Baillie, nephew of William Hunter and Physician to George III. Our readers will remember the physician's cane mentioned in the history of Bristol Royal Infirmary, a cane which was the favourite argument of the physician to whom it belonged.

### THE SERVANTS' HOSPITAL BED.

What a sign of the times it is that domestic servants are becoming a class which can organise an appeal among its members to endow a bed for itself in a voluntary hospital. We welcome the change, and still more that it has followed, not preceded, the policy of at least a few hospitals to provide a proper home for the domestic staff. Aptly enough the appeal is in connection with the Garrett Anderson Hospital Fund. Six hundred pounds were raised from domestic servants, and it was desired to secure the full sum by the fancy dress dance recently held at the Artists' Rifles Drill Hall in Euston Road. Not only did the butlers and other servants from various big houses attend, but also their mistresses. Miss Morris, the appeal secretary, made the arrangements, and we congratulate her in drawing together for a conclusive object members of an occupation who have been far too much isolated in the past in dark basements and high attics, and have spent the best of their youth ascending and descending ladder-like stairs. For the Victorian town house was a triumph of mean design to which the limbs and lives of many young men and women were needlessly and cruelly sacrificed. This, we believe, will be the first "Domestic Servants' Bed" yet endowed.

### THE TRUE GENEROSITY.

It is always pleasant to see the secretary of anything suitably recognised, for the work is often arduous, the detail persistent, the reward mainly that nothing has gone wrong! We think, then, that Mr. C. H. Armitage, C.B.E., Hon. Secretary and Hon. County Director for the Derbyshire Branch of the Red Cross Society, must have been pleased with his recent presentation, for in making it Colonel Brooke Taylor said that he believed Mr. Armitage was the only amateur whom he had ever met who never flinched for five years at any duty, however objectionable it might be. He gave his whole time to the work. Cottage hospitals have long been favoured by similar ungrudging service on the part of men who have to fit the work in with their professional engagements. To be generous with one's time is more exacting than to be generous with one's money, and perhaps because it is the truer gift it is less publicly recognised.

### HOSPITAL OFFICERS' ASSOCIATION.

The members of the Incorporated Association of Hospital Officers, in number about fifty, visited King's College Hospital on the 6th inst., and were shown over the building by the Sister, Matron, the Secretary, and other officers. Many of the special departments were visited, and the members were much interested to hear that six infants had been born in the maternity department on the day of the visit. After the inspection the annual service of the Association was held in the chapel, the service being choral, and an appropriate address being given by the Rev. R. V. Galer. A very instructive and enjoyable afternoon was concluded by tea in the board room.

### THE CORRUPTION OF CHARITY.

The Kent County Ophthalmic Hospital at Maidstone has had the remarkable experience of raising within twelve months the number of its weekly subscribers from 1,500 to 18,000. Mr. E. Rumbelow appears to be largely responsible for this feat. Is the hospital wise, however, to guarantee free treatment in sickness to every weekly subscriber, whatever his wages, and to the members of his family if wholly dependent on him? The hospital has done so, and the men's delegates were insistent. Consequently they have reduced the men whom they represent to the level of tradesmen driving a hard bargain, and the attitude of insistence that no man's wages should be taken into consideration is degrading for the better-paid men. The proper cause was to give to the delegates a number of tickets in proportion to the sum subscribed. These could then be handed to those in need; but it should have been made clear that voluntary hospitals are not commercial concerns, and give to no subscriber a right of "entry." A person subscribes to hospitals that other people may benefit in time of need, and why a weekly subscription should carry the degrading privilege of right of entry we do not know; and we doubt if the men appreciate the indignity of the position into which they have been placed. The right of entry to a hospital belongs to the sick, and the attempt to purchase this right is a dishonour.

### CINEMATOGRAPH PATIENTS! !

THE Birmingham Cinema Hospital Sunday seems to have been successful, and, so far as we know, to have met with no hostile criticism. This may have been due to the fact that all the picture-houses (save one) combined for the purpose, or to the fact, which is to be inferred, that the net proceeds were given to the hospitals without deduction for expenses. The plan originated also with the industry, and is to become its annual gift. A benevolent fund for those engaged in the trade is also contemplated. It is curious to remember that Birmingham, where the Sunday Fund was started, is also the first place to found a Cinematograph Fund for hospitals. It is not less curious to note that the first hospital fund was started as the expression of a gospel. The day was devoted to an exposition of personal service, and one of its expressions, but one only, was the money subscribed. With the passage of time, the collection of money for hospitals threatens to become a business, and in consequence any means of raising money is resorted to. People pay to see the show or visit the bazaar, but it is the show and not the object of the show which is allowed to interest them. A curious symptom of the time is the statement of Lady Astor, M.P., that charity " hurts the giver because it gives him a smug sense of self-righteousness." This statement was made during her visit to the Hospital for Sick Children in Great Ormond Street, and " formed part " of the appeal! Since Lady Astor was not interrupted by the audience, we presume that she echoed its opinions. No wonder, then, the voluntary hospitals do not get their money if this is their attitude to the cause that they represent.

### A BAD EXAMPLE FROM LIVERPOOL.

IT does not read well that Liverpool employers have refrained from contributing to the Lord Mayor's appeal for Liverpool hospitals until they have assured themselves that the workmen are contributing in proportion. Far too often the workmen have given a lead in this respect, while employers have been content with the cheap insurance of a casual cheque and the sight of their names high in the list of contributors. It is the latter's duty to give a lead to their men, not to cringe behind them in this matter. It is theirs to learn also that a big business like a large property has duties, and that one of these duties is to give to charity. The profit-monger has always been a despicable figure, and the bad blood which he creates between classes inevitably produces the suspicion and distrust that is the fertile soil of the agitator and the theorist. Leadership is to him who leads, and if the Liverpool employers have not the decency or public spirit to set an example of generosity and then to invite their men to follow it, it is a poor lookout for the industrial atmosphere of the town.

### A REALLY ANONYMOUS DONOR.

THE anonymous donor, let us assume it is one person, has done well in his three gifts of £20,000 each to the Manchester Royal Infirmary, the Liverpool Royal Infirmary, and to Guy's Hospital. The gift is really anonymous, because the only people capable of tracing the donor are Messrs. Glyn, Mills, Currie and Co., through whom each of the payments was made.

### DR. MONTESSORI AT MANCHESTER.

THOSE of our readers who enjoyed the lecture of Dr. Montessori which recently appeared in THE HOSPITAL, will be interested to know that she is opening the extension of the Greengate Dispensary and Day Nursery at Salford, Manchester, next month. She will secure a good audience, and the committee of the home is to be congratulated in having secured so distinguished a speaker.

### THE SALE OF ONTARIO HOSPITAL.

THE Ministry of Pensions has purchased for £80,000 from the Government of Ontario the Ontario Military Hospital, at Orpington. It was the biggest Canadian war hospital in this country, accommodated 2,000 patients, and was built specially for the purpose. It will be used for the treatment of convalescent and disabled soldiers when they come from the War Office to the care of the Ministry.

### BALTIC AND LLOYD'S AMBULANCE FUNDS.

To wind up the " Baltic " Ambulance Fund a meeting was held on November 25, at the Merchant's Hall of the Baltic Mercantile and Shipping Exchange. There was a balance of over £13,000 in hand, and it was resolved to give this away as follows:—Charing Cross Hospital, the London Hospital, St. Thomas's Hospital, The Dreadnought Hospital, St. Andrew's Hospital, and four other charities £1,000 each, and the Seamen's Orphanage and St. Bartholomew's Hospital £2,000 each, the last-named gift being in memory of Mr. Ford Clark and Mr. F. W. Davis, engineer to the Baltic Ambulance Unit. Mr. Philip Runciman, who was in the chair, mentioned that the Ambulance had carried nearly 40,000 cot cases, and the night transport about 160,000 men, mostly soldiers who were coming from or going on leave from the various stations, when there were no Tube trains or omnibuses running. Thus finishes a very useful organisation, which has been carried through without getting into debt, and which, after carrying out its functions in a perfectly efficient way, is able to help very substantially some of the more important charities within the Metropolitan area.

### THIS WEEK'S DRUG MARKET.

IN spite of the near approach of Christmas the drug market is busy, especially the export side of it. It is the big demand for drugs from all over the world that is the main cause of the continued upward tendency of prices. Business in quinine has not yet settled down, and it is still difficult to quote a stable price. Menthol goes from one high point to another still higher, and much the same applies to Japanese peppermint oil. Camphor also continues to move upwards in value. Mercurials have again advanced in price following on the rise in quicksilver. Cassia oil is dearer. Cocaine is still in short supply. Ergot of rye is again higher in price. Gum tragacanth is dearer. The advanced quotation for resorcin is well maintained. Thymol is dearer. Salicylates have an upward price tendency.

